# Identifying biomarkers of endoplasmic reticulum stress and analyzing immune cell infiltration in chronic obstructive pulmonary disease using machine learning

**DOI:** 10.3389/fmed.2024.1462868

**Published:** 2024-11-22

**Authors:** Shuaiyang Zhang, Hangyu Duan, Jun Yan

**Affiliations:** ^1^Dongzhimen Hospital of Beijing University of Chinese Medicine, Beijing, China; ^2^Xiyuan Hospital, China Academy of Traditional Chinese Medicine, Beijing, China

**Keywords:** chronic obstructive pulmonary disease, endoplasmic reticulum stress, machine learning, immune cell infiltration, BCHE, CBY1, EDEM3

## Abstract

**Background:**

Endoplasmic reticulum stress (ERS) is a crucial factor in the progression of chronic obstructive pulmonary disease (COPD). However, the key genes associated with COPD and immune cell infiltration remain to be elucidated. Therefore, this study aimed to identify biomarkers pertinent to the diagnosis of ERS in COPD and delve deeper into the association between pivotal genes and their possible interactions with immune cells.

**Methods:**

We selected the genetic data of 189 samples from the Gene Expression Omnibus database, including 91 control and 98 COPD samples. First, we identified the differentially expressed genes between patients with COPD and controls and then screened the ERS genes associated with COPD. Second, 22 core ERS genes associated with COPD were screened using the Least Absolute Shrinkage and Selection Operator (LASSO) regression model and Support Vector Machine Recursive Feature Elimination (SVM-RFE), and the predictive effects of the screened core genes in COPD were evaluated. Third, we explored immune cell infiltration associated with COPD and conducted an in-depth analysis to explore the possible connections between the identified key genes and their related immune cells.

**Results:**

A total of 66 differentially expressed endoplasmic reticulum stress–related genes (DE-ERGs) were identified in this study, among which 12 were upregulated and 54 were downregulated. The 22 key genes screened were as follows: AGR3, BCHE, CBY1, CHRM3, CYP1B1, DCSTAMP, DDHD1, DMPK, EDEM3, EDN1, FKBP10, HSPA2, KPNA2, LGALS3, MAOB, MMP9, MPO, MTTP, PIK3CA, PTGIS, PURA, and TMCC1. Their expression was significantly different between COPD and healthy samples, and the difference between the groups was significant. Receiver operating characteristic curve analysis revealed that CBY1 (area under the curve [AUC] = 0.800), BCHE (AUC = 0.773), EDEM3 (AUC = 0.768), FKBP10 (AUC = 0.760), MAOB (AUC = 0.736), and MMP9 (AUC = 0.729) showed a strong ability to distinguish COPD samples from normal samples. Immune cell infiltration results associated with the three key genes were also obtained.

**Conclusion:**

The insights of our study have the potential to present new evidence for exploring emerging diagnostic signs of COPD while also contributing to a better understanding of its developmental mechanisms.

## 1 Introduction

Among the myriad of chronic lung afflictions, chronic obstructive pulmonary disease (COPD) is highly prevalent in the modern era; it is typically characterized by irreversible airflow limitations due to airway narrowing and is usually diagnosed in patients with chronic bronchitis and emphysema when they develop persistent airflow limitation on pulmonary function testing (1, 2). Reportedly, the prevalence of COPD among people over 40 years of age is 13.6% in China and up to 15–20% in Europe (3, 4). As one of the three most common causes of death, COPD is often difficult to cure, and its clinical symptoms are often characterized by nocturnal exacerbation of cough, sputum production, dyspnea, and wheezing, which greatly affect patients’ quality of life (5). Each acute exacerbation substantially increases the cost of treatment, creating a huge financial burden for individuals, families, and the country as well as implying a high mortality risk (6). Notably, COPD often comorbidly occurs with other diseases such as osteoporosis, anemia, malnutrition, peripheral vascular disease, and coronary artery disease (7, 8). The pathogenesis of COPD is associated with many factors, such as inflammatory cell activation, protease/antiprotease imbalance, oxidative stress, genetic/epigenetic modifications, and endoplasmic reticulum stress (ERS) (9–12). Therefore, early identification of biomarkers for COPD is necessary.

The endoplasmic reticulum (ER) is an important intracellular structure that plays a key role in protein synthesis, folding, clearance, assembly, translation, and modification (13). ERS is triggered by changes in the environment, such as temperature and nutrient conditions, leading to an imbalance in ionic and protein homeostasis within the cell and resulting in massive aggregation of misfolded or unfolded proteins (14). To restore the ER and protein homeostasis, cells activate the unfolded protein response (UPR), an adaptive signaling pathway activated by inositol-requiring protein-1, protein kinase RNA-like endoplasmic reticulum kinase, and activating transcription factor-6 (15). If homeostasis cannot be restored, excessive ERS leads to ER damage and the activation of pro-apoptotic systems, which are increasingly evidenced as potentially important mechanisms in the development of various diseases (16). Further, excessive ERS leads to apoptosis of lung endothelial cells, which is closely related to COPD development (17).

Machine learning, differentiated from traditional statistical methods that process complicated data and complex problems, has gradually gained favor in various fields (18). Currently, in the field of medicine, machine learning is widely used in healthcare, diagnosis, predictive model construction, image processing, bioinformatic analysis, and many other aspects (19). Machine learning can organize redundant and numerous medical data quickly and with minimal cost to analyze the relationships between various variables and factors and help us more clearly understand the underlying mechanisms of disease development (20). Therefore, the use of machine learning algorithms to explore disease biomarkers is important for identifying new targets for treating diseases.

In this study, we identified 66 ER stress–related genes (ERGs) by analyzing the association between the extracted genes related to ERS and the differentially expressed genes between individuals with COPD. To better predict the occurrence of COPD, we identified the major genes associated with COPD and immune cell infiltration using machine learning algorithms. This study has the potential to shed more light on the role of ERS in the pathogenesis of COPD, concurrently suggesting novel strategies for its clinical management.

## 2 Materials and methods

### 2.1 Data collection

We downloaded public microarray data, GSE76925 and GSE38974, containing clinical information on COPD and normal lung tissues, from the Gene Expression Omnibus database.^1^ They are well annotated for genetic data. The GSE38974 dataset, derived from the GPL4133 platform, included 23 COPD lung tissue samples and 9 normal samples. The GSE76925 dataset, derived from the GPL10558 platform, included 111 COPD lung tissue samples and 40 normal samples. Empirical Bayes methods can eliminate batch effects in the analysis of microarray expression data by adjusting the standard error of the estimated multiplicative changes (21). This study used empirical Bayes methods to eliminate group effects and ultimately merged and normalized the data sets into 98 COPD samples and 91 normal tissue samples. We screened 1,350 ERGs with relevance scores >5 from the GeneCards database.

### 2.2 Screening and functional enrichment analysis of ERGs

This study used the merge function to combine the two datasets into a single metadata cohort and employed the combat function to eliminate batch effects. Differentially expressed endoplasmic reticulum stress–related genes (DE-ERGs) between COPD and healthy samples were determined using the Limma package in R. The screening thresholds for differentially expressed genes (DEGs) were set as |Log2FC| > 1, *p* < 0.05, and a false discovery rate (FDR) < 0.05. FDR measures the proportion of false discoveries among a set of hypothesis tests that are deemed significant. Subsequently, DE-ERGs were identified by intersecting the DEG list. To determine the biological significance of DE-ERGs in COPD, we conducted enrichment analyses in Gene Ontology (GO) and Kyoto Encyclopedia of Genes and Genomes (KEGG) using the ClusterProfiler package (22). The adjusted significance level was set at *p* < 0.05. Additionally, we conducted Disease Ontology (DO) enrichment analysis of DE-ERGs using the “clusterProfiler” and DOSE packages.

### 2.3 Selection of feature genes

The selection of feature genes was performed using two machine learning algorithms: Least Absolute Shrinkage and Selection Operator (LASSO) regression model and Support Vector Machine Recursive Feature Elimination (SVM-RFE). During the fitting of the generalized linear model, LASSO was used for dimensionality reduction to accomplish variable selection (23). LASSO evaluation was performed using the “glmnet” software, with the penalty parameter set to 10-fold cross-validation. SVM-RFE, on the other hand, implemented small-scale learning and sample prediction through “conductive inference,” effectively reducing common regression and classification issues.

### 2.4 CIBERSORT analysis

The CIBERSORT computational method^2^ is a deconvolution algorithm that analyzes complex gene expression profiles to infer the composition of immune cells (24). Using the CIBERSORT algorithm, we identified the immune responses of 22 types of immune cells and evaluated the relationship between these immune cells and the expression of key genes in normal and COPD samples. This approach helped clarify correlations between various immune cells.

### 2.5 Statistical analysis

We used *t*-tests to compare gene expression between COPD and adjacent healthy samples. To evaluate the classification performance of key genes in COPD and healthy samples, receiver operating characteristic (ROC) curves and areas under the curve (AUC) were calculated using the “pROC” package in R. Statistical analyses were conducted using R version 4.4.0. Differences were considered statistically significant at *p < 0.05, **p < 0.01, and ***p < 0.001.

## 3 Results

### 3.1 Screening and functional enrichment analysis of DE-ERGs

**FIGURE 1:**
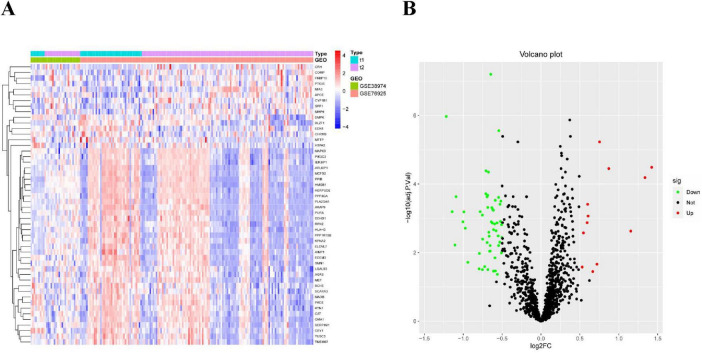
**(A)** Heatmap of expression patterns of ERGs showing differential expression in COPD; **(B)** volcano plot of expression patterns of ERGs showing differential expression in COPD.

**FIGURE 2:**
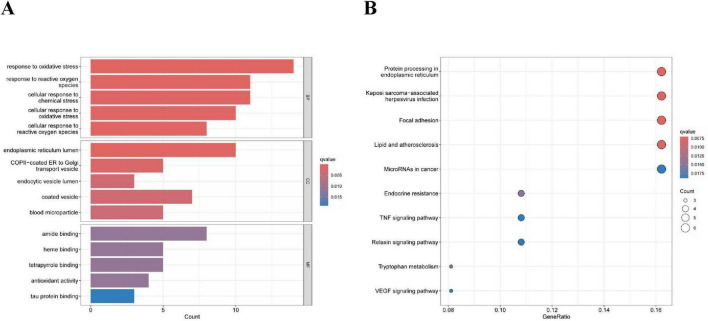
GO analysis **(A)** and KEGG analysis **(B)** for the 66 DE-ERGs.

After screening, we identified a total of 66 DE-ERGs. We performed heatmap analysis of these genes (Figure 1A). Among these, 12 genes were upregulated and 54 were downregulated (Figure 1B). Next, we conducted an enrichment analysis of the biological functions of these genes (GO and KEGG analyses). Our findings revealed that the 66 DE-ERGs predominantly participated in biological processes, including the reaction to oxidative stress and the reaction of cells to reactive oxygen species. Cellular components were primarily enriched in the ER lumen, coated vesicles, and blood microparticles. In the molecular function category, the genes were mainly enriched in amide and heme binding (Figure 2A). KEGG analysis indicated that DE-ERGs were significantly enriched in pathways, including the TNF, VEGF, and relaxin signaling pathways (Figure 2B).

### 3.2 Selection of feature genes

**FIGURE 3:**
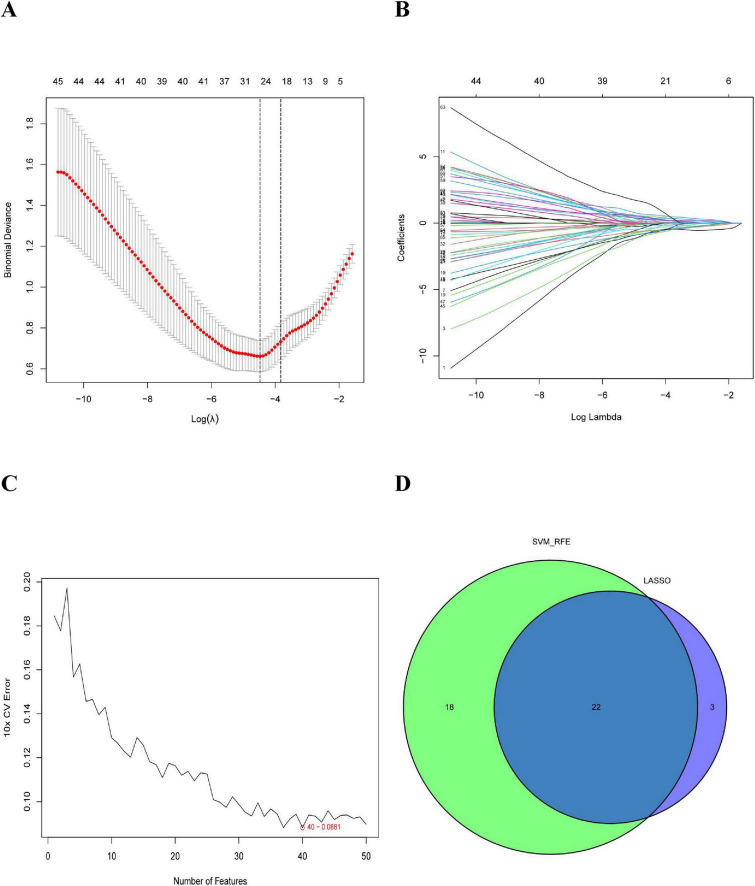
Selection of COPD-related signature genes: **(A)** graph of signature tuning screening in the LASSO model; **(B)** path diagram of the LASSO model; **(C)** graph of biomarker screening by the SVM-RFE algorithm; and **(D)** venn diagram showing the 22 diagnostic biomarkers jointly identified by LASSO and SVM-RFE.

In this study, we used the LASSO regression and SVM-RFE algorithms to screen for potential biomarkers. The LASSO regression algorithm identified DEGs and determined 25 variables as diagnostic markers for COPD. The SVM-RFE algorithm further screened 40 features of these DEGs (Figure 3). Finally, 22 features identified by both algorithms were selected: AGR3, butyrylcholinesterase (BCHE), Chibby1 (CBY1), CHRM3, CYP1B1, DCSTAMP, DDHD1, DMPK, ER-degradation alpha-mannosidase–like protein 3 (EDEM3), EDN1, FKBP10, HSPA2, KPNA2, LGALS3, MAOB, MMP9, MPO, MTTP, PIK3CA, PTGIS, PURA, and TMCC1. These 22 genes might play significant roles in the occurrence and development of COPD.

### 3.3 Expression and diagnostic significance of feature genes in COPD

**FIGURE 4:**
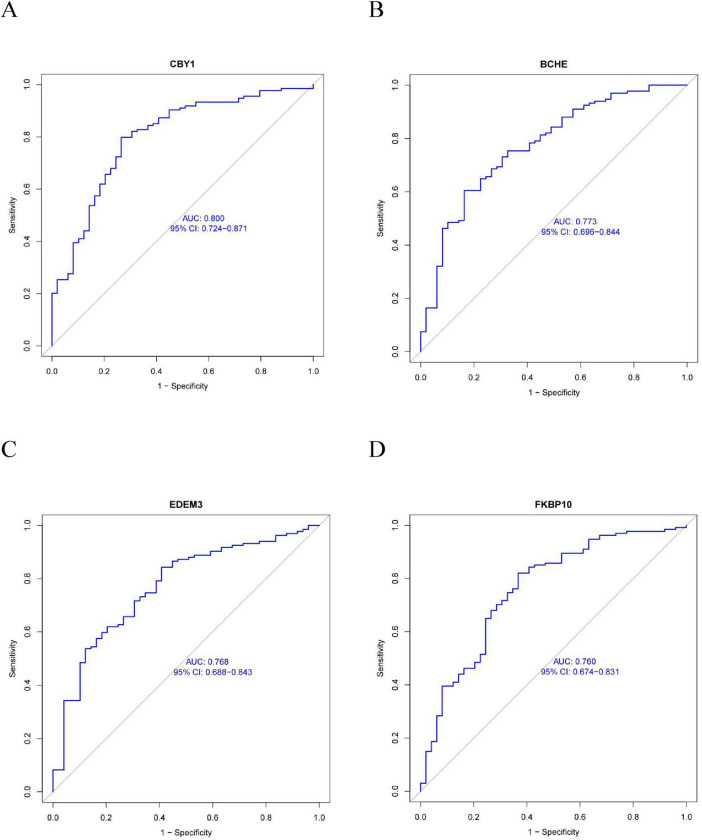
Diagnostic significance of characterized genes in COPD: **(A)** CBY1; **(B)** BCHE; **(C)** EDEM3; **(D)** FKBP10.

**FIGURE 5:**
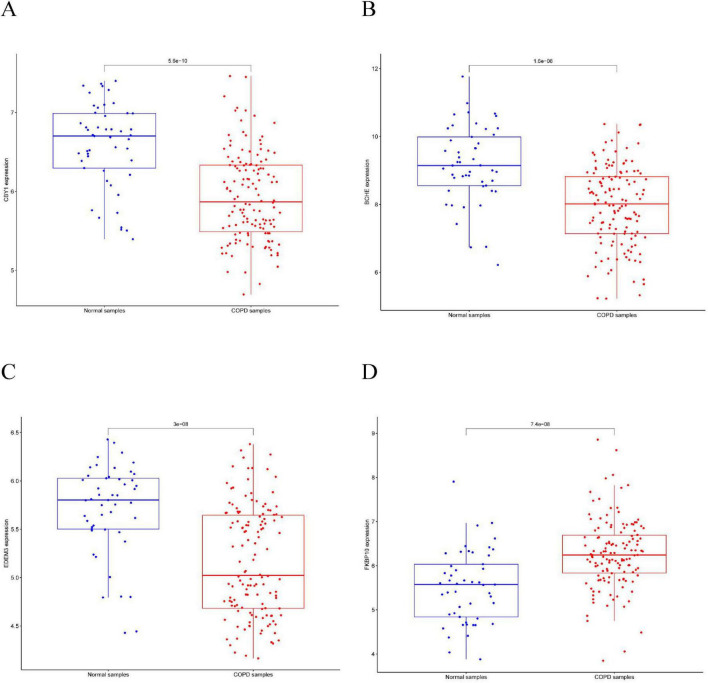
Expression of characterized genes in COPD: **(A)** CBY1; **(B)** BCHE; **(C)** EDEM3; **(D)** FKBP10.

This study compared the expression levels of AGR3, BCHE, CBY1, CHRM3, DDHD1, DMPK, EDEM3, EDN1, HSPA2, KPNA2, LGALS3, MAOB, PIK3CA, PURA, and TMCC1 between COPD and control groups and evaluated their predictive abilities. As shown in Figure 4, the AUC values for CBY1 (AUC = 0.800), BCHE (AUC = 0.773), EDEM3 (AUC = 0.768), and FKBP10 (AUC = 0.760) exceeded 0.75, indicating a good predictive ability for COPD. As shown in Figure 5, the expression levels of CBY1, BCHE, EDEM3, and FKBP10 differed significantly between the COPD and control groups. Specifically, FKBP10 expression was significantly elevated in the COPD samples, whereas the expression levels of CBY1, BCHE, and EDEM3 were significantly reduced in COPD samples.

### 3.4 Immune infiltration analysis

**FIGURE 6:**
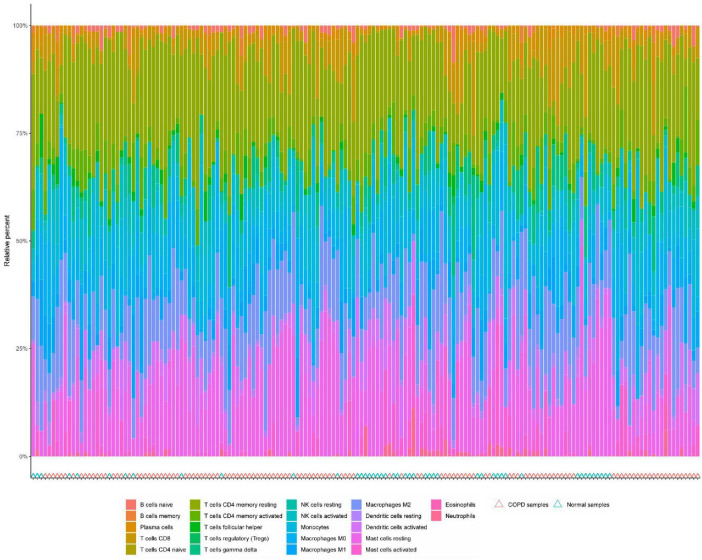
Percentage of 22 immune cells identified by the CIBERSORT algorithm.

**FIGURE 7:**
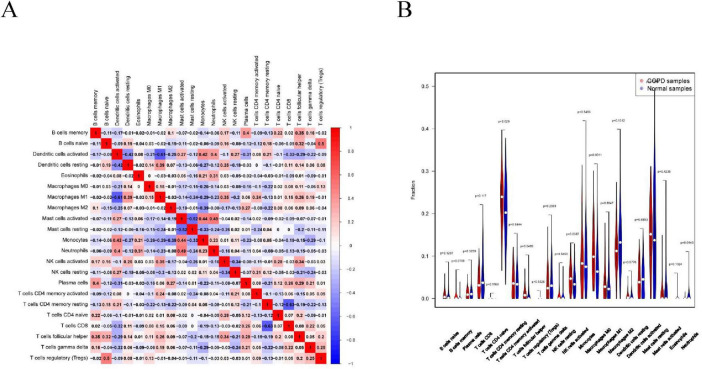
Immune cell infiltration analysis: **(A)** association between the immune system and COPD. Red color indicates a positive association while blue color indicates a negative association. Darker colors indicate a stronger relationship; **(B)** graph of the difference in immune cell infiltration between the COPD group and the healthy control group.

**FIGURE 8:**
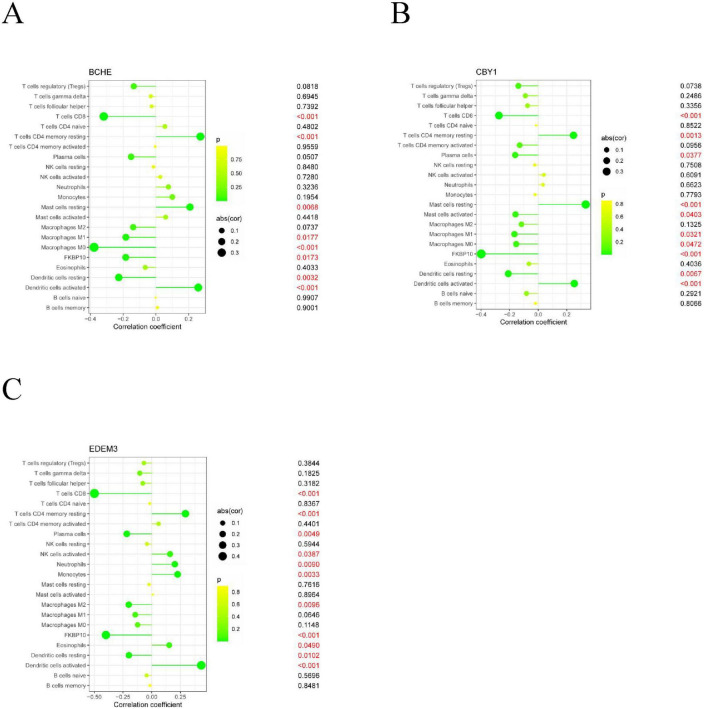
Association analysis between important characterized genes and immune infiltrating cells: **(A)** BCHE; **(B)** CBY1; **(C)** EDEM3.

Immune cell infiltration is an important predictor of COPD prognosis. We compared 22 immune cell subpopulations in the patient population with COPD (Figure 6). Correlation heat maps and violin plots were created to visualize the data. The heatmaps showed the proportional distribution of 22 different immune cell types in COPD, revealing significant patterns. The results indicated a strong positive correlation between naïve B cells and regulatory T cells, a strong negative correlation between activated dendritic cells and M1 macrophages, a significant negative correlation between activated mast cells and resting mast cells, and a significant positive correlation between activated mast cells and neutrophils (Figure 7A). The Wilcoxon test was used to evaluate significant differences in immune cell infiltration between COPD and control samples. The results showed that compared with non-COPD individuals, COPD patients had significantly higher levels of resting CD4 memory T cells, T follicular helper cells, activated NK cells, and M0 macrophages (Figure 7B). We further explored the associations between three important biomarkers (BCHE, CBY1, and EDEM3) and various immune cells (Figure 8). Correlation analysis showed that BCHE significantly negatively correlated with T cells CD8, macrophage M1 (MM1), macrophage M0 (MM0), FKBP10, and dendritic cell resting (DCR), whereas it significantly positively correlated with resting CD4 memory resting (CD4MR), mast cell resting (MCR), and dendritic cell activation (DCA) T cells. CBY1 significantly negatively correlated with T cells CD8, plasma cells (PC), activated mast cells (MCA), MM1, MM0, FKBP10, and DCR, whereas it significantly positively correlated with CD4MR, MCR, and DCA. EDEM3 significantly negatively correlated with T cells CD8, PC, macrophages (M2), FKBP10, and DCR, whereas it significantly positively correlated with CD4MR, activated NK cells, neutrophils, monocytes, eosinophils, and DCA. Our findings suggest that these key genes influence the occurrence and development of COPD by regulating immune cell infiltration.

We further explored the association between three important biomarkers, BCHE, CBY1, and EDEM3, and various immune cells (Figure 8). The results of the correlation analysis showed that there were significant negative correlations between BCHE and T cells CD8, MM1, MM0, FKBP10, and DCR and a significant negative correlation with dormant CD4MR, MCR, and DCA. There was a significant negative correlation between CBY1 and CD8, PC, MCA, MM1, MM0, FKBP10, and DCR and a significant positive correlation between CBY1 and CD4MR, MCR, and DCA. EDEM3 expression significantly correlated with CD8, MM2, FKBP10, and DCR T cells. There was a significant negative correlation between EDEM3 and CD8, PC, MM2, FKBP10, and DCR T cells and a significant positive correlation with CD4MR, activated NK cells, neutrophils, monocytes, eosinophils, DCA. Our results suggest that these key genes influence COPD development by regulating immune cell infiltration.

## 4 Discussion

The typical pathological changes in COPD mainly include airway pathological changes (such as degenerative necrosis of airway epithelial cells, increased airway mucus secretion, squamous epithelial cell hyperplasia, airway remodeling, thickening of small airway walls), inflammatory responses (such as inflammatory cell infiltration and septic inflammation), and lung structural changes (such as emphysema and destruction of lung tissue structures), all of which accelerate lung aging, often resulting in lung failure, dyspnea, and death (25, 26). However, the pathogenesis of COPD requires further investigation. Currently, studies analyzing a correlation between ERS and COPD are underway. One study found that cigarette smoke extract exposure caused mouse lung endothelial cells to activate the UPR, leading to enhanced eIF2α phosphorylation, which in turn enhanced susceptibility to lung endothelial cell apoptosis and emphysema (27). Tao et al. screened six potential ERGs in COPD by analyzing databases and blood samples from the COPD patient group and normal blood samples from the COPD patient and control groups. They found that the expression of key ERS genes (APAF1, BAX, PPP1R3C, PTPN1, and STC2) was significantly elevated in COPD patients, accompanied by a significant decrease in lung function–related indices, verifying the reliability of the results (28). The aim of this study was to compare the differences in the expression of ERGs between COPD patients and healthy controls and investigate the potential role of these ERGs in COPD development and immune cell infiltration.

In this study, three characterized genes (BCHE, CBY1, and EDEM3) were identified using machine learning algorithms combined with ROC analysis. BCHE is a serine hydrolase widely found in various organs and tissues of the human body, such as the viscera, blood, skin, muscle, and brain. It is most abundant in the blood and liver and promotes the hydrolysis of acetylcholine to restore resting cholinergic neurons. As early as 60 years ago, researchers used extracted and purified BCHE in the clinic, and its effects included detoxification, hydrolysis of acetylcholine, promotion of fat metabolism, and removal of polyproline-rich peptides (29). A study by Anes et al. found that in patients with COPD, BCHE activity significantly increased in the lungs, did not significantly correlate with smoking, and was accompanied by a high expression of markers of oxidative damage to proteins, such as total protein carbonyls and advanced oxidized protein products (30). However, the results of the study by Sicinska et al. were different, as they analyzed blood samples from 30 patients with COPD and 18 healthy subjects in a controlled manner and found that BCHE activity was significantly reduced in the blood of patients with COPD, which was accompanied by an increase in lipid peroxidation and a decrease in total antioxidant capacity (31). Gu et al. explored BCHE activity through an *in vitro* analysis of prostate cancer–associated cell cultures to investigate the effect of BCHE in prostate cancer, which showed biphasic alterations, that is, downregulation in the early stage and upregulation in the later stage (32). Zengin et al. found that BCHE expression levels were reduced in lung cancer patients (33). Therefore, BCHE could potentially serve as a diagnostic or prognostic biomarker in ERS-associated COPD.

CBY1, also known as β-cyclin antagonist, is a protein-coding gene that competes with transcription factors for binding to β-cyclin, a transcriptional activator and oncoprotein involved in the development of several cancers, to inhibit its mediated transcriptional activation. Reportedly, CBY1 is significantly downregulated in patients with chronic granulocytic leukemia, which can be upregulated by relevant inhibitors to induce ERS-associated UPR, which promotes β-cyclin inactivation, leading to apoptosis and cell death to eradicate BCR-ABL1 + hematopoiesis for therapeutic purposes (34). In addition, mutations and the downregulation of CBY1 have been associated with a variety of diseases such as ciliopathy characterized by Joubert syndrome (35), pancreatitis (36), and colon cancer (37). However, there are fewer relevant studies on the relationship between CBY1 and COPD, and the role of CBY1 in the inflammatory response, alteration of airway endothelial cells, apoptosis, oxidative stress, and other processes in the pathogenesis of COPD needs to be further explored. EDEM3 is a soluble homolog of ER degradation–enhancing alpha-mannosidase–like proteins that promote ER degradation of misfolded glycoproteins and participate in mannose pruning (38). Reportedly, the inhibition of EDEM3 expression can reduce triglyceride levels *in vivo* (39) and the radioresistance of prostate cancer cells (40). Extensive research on the relationship between EDEM3 and COPD is warranted.

Previous studies on identifying ERS-related COPD diagnostic markers and analyzing related immune infiltration have been insufficient. This study is thus relevant because it extracted and analyzed data from publicly available databases, delineated relevant markers, and provided directions for further in-depth studies. This study has some limitations. First, owing to limited funding, we are unable to validate the accuracy of these key genes as biomarkers at this time, and we will later conduct immunoinfiltration and other relevant clinical trials to validate the results and conclusions of the present experiments. Second, the performance of LASSO and SVM-RFE in large-scale or updated datasets is limited as well as their validation and generalizability in bioinformatics analysis—these must be addressed in future research.

In conclusion, using machine learning algorithms combined with immune infiltration analysis, we screened for ERGs associated with COPD and analyzed the correlation between key genes and immune cell infiltration. The findings of this study have important clinical implications for the diagnosis, treatment, and prognosis of COPD. However, the complex mechanisms of key genes in COPD identified in this study need to be explored further.

## Data Availability

The original contributions presented in this study are included in this article/Supplementary material, further inquiries can be directed to the corresponding author.

## Footnotes

1
https://www.ncbi.nlm.nih.gov/geo/

2
http://cibersort.stanford.edu/
